# Prevalence and risk factors of cryptosporidiosis among children under 5 years of age with diarrhea in internally displaced persons camps in Mogadishu, Somalia: a cross-sectional study

**DOI:** 10.1016/j.ijregi.2026.101044

**Published:** 2026-10-16

**Authors:** Bashir Abdirahman Guled, Abdifetah Ibrahim Omar, Hussein Ibrahim Gabow, Anwar Abdullahi Adam, Ifrah Ahmed Warsame, Hafza Jama Hayow, Kauther Mohamed Omar, Omar Osman Mohamed, Mohamed Khalif Abdulle, Ahmed Hassan Said, Ahmed Saed Hussein

**Affiliations:** Faculty of Medicine and Health Sciences, Jamhuriya University of Science and Technology, Mogadishu, Somalia

**Keywords:** Cryptosporidiosis, *Cryptosporidium*, Diarrhea, Children under 5, Internally displaced persons, Somalia

## Abstract

**Objectives:**

Cryptosporidiosis is an important cause of diarrheal disease among young children, particularly in resource-limited settings and displaced populations. However, evidence on *Cryptosporidium* infection among children living in internally displaced persons (IDPs) camps in Somalia remains limited. This study aimed to determine the prevalence of *Cryptosporidium* infection and identify associated factors among children under 5 years of age with diarrhea who live in IDPs camps in Mogadishu, Somalia.

**Methods:**

A cross-sectional study was conducted from December 2022 to August 2023 among 384 children under 5 years of age with diarrhea residing in IDPs camps in the Daynile District of Mogadishu, Somalia. Sociodemographic, clinical, and hygiene-related data were collected using a structured questionnaire. Stool samples were examined for *Cryptosporidium* oocysts using the modified Ziehl–Neelsen staining technique. Chi-square tests and logistic regression were used to assess factors associated with the infection.

**Results:**

*Cryptosporidium* oocysts were detected in 23 of 384 children, giving a prevalence of 6.0%. Low, moderate, and high oocyst loads were observed in 10 (2.6%), eight (2.1%), and five (1.3%) children, respectively. In the multivariable logistic regression analysis, primary caregiver education was associated with lower odds of infection compared with secondary education (adjusted odds ratio = 0.08; 95% confidence interval: 0.01-0.75; *P* = 0.027). Seeking medical care for diarrheal illness was also associated with lower odds of infection (adjusted odds ratio = 0.28; 95% confidence interval: 0.09-0.90; *P* = 0.032). No statistically significant associations were observed between the child’s sex or caregiver occupation and infection.

**Conclusion:**

*Cryptosporidium* infection was detected among children with diarrhea living in IDPs camps in Mogadishu. Caregiver education and health care–seeking behavior were associated with infection, although the cross-sectional design does not establish causality. Strengthening water, sanitation, and hygiene services; improving access to health care; and using more sensitive diagnostic methods may help to improve the prevention and detection of cryptosporidiosis in these settings.

## Highlights

• This study provides the first evidence of cryptosporidiosis among children under 5 years of age in internally displaced persons camps in Mogadishu, Somalia.
• The prevalence of *Cryptosporidium* infection among children with diarrhea was 6.0% in this displaced population.
• Male sex and caregiver unemployment were significantly associated with increased odds of infection.
• Access to health care services was associated with lower odds of *Cryptosporidium* infection.
• Findings highlight the need for integrated water, sanitation, hygiene, and health care interventions in internally displaced persons settings.

## Introduction

Cryptosporidiosis is an enteric disease caused by the protozoan parasite *Cryptosporidium*, which infects a wide range of vertebrate hosts, including humans [1]. Since the 1980s, cryptosporidiosis has been recognized as a major cause of acute and chronic diarrhea, particularly among young children and immunocompromised individuals, and is now considered an important global public health concern [2]. Worldwide, *Cryptosporidium* is one of the leading causes of waterborne disease outbreaks and ranks as the second most common cause of diarrheal mortality in children under 5 years of age, after rotavirus [3]. In low- and middle-income countries, cryptosporidiosis is also associated with malnutrition, growth stunting, and increased childhood mortality [4].

Transmission of cryptosporidiosis occurs primarily through the fecal–oral route, either by direct person-to-person contact or indirectly through the ingestion of contaminated water or food [5]. The parasite produces environmentally resistant oocysts that can survive for long periods in water, soil, and on surfaces contaminated with human or animal feces [6]. Zoonotic transmission is also common, particularly in settings where humans live in close proximity to livestock. These characteristics make *Cryptosporidium* especially difficult to control in overcrowded environments with limited access to safe water, sanitation, and hygiene (WASH) facilities [7].

Diagnosis of cryptosporidiosis can be performed using several methods, including conventional microscopy, antigen detection assays, and molecular techniques such as polymerase chain reaction. Modified Ziehl–Neelsen staining remains widely used in resource-limited settings because of its low cost and simplicity; however, this method has lower sensitivity than antigen-based or molecular assays and does not allow species differentiation [3,8,9]. As a result, cryptosporidiosis is frequently underdiagnosed and underreported in many developing countries, particularly where laboratory capacity is limited [10].

The burden of cryptosporidiosis is disproportionately high in sub-Saharan Africa, which accounts for a large share of global childhood deaths from diarrheal diseases. Studies from countries such as Cameroon, Ethiopia, Sudan, Tanzania, and Nigeria have reported variable prevalence rates of cryptosporidiosis among children with diarrhea, reflecting differences in environmental conditions, diagnostic methods, and study populations [11,12]. Despite this regional burden, surveillance data remain limited in many fragile and conflict-affected settings [13,14]. *Cryptosporidium* infection is an important opportunistic infection among people living with human immunodeficiency virus (HIV), particularly those with advanced immunosuppression. In these individuals, the infection may cause persistent and severe diarrhea, contributing to increased morbidity and mortality. Although the prevalence of HIV in Somalia is relatively low compared with many countries in sub-Saharan Africa, HIV remains an important consideration in the epidemiology and clinical impact of cryptosporidiosis [[15], [16], [17]].

In Somalia, children under 5 years of age living in overcrowded internally displaced persons (IDPs) camps are especially vulnerable to enteric infections due to inadequate WASH infrastructure [18]. However, to date, there is a marked lack of data on cryptosporidiosis in Somalia, and no previous studies have specifically investigated its prevalence or associated risk factors among children residing in IDPs camps.

Therefore, this study aimed to determine the prevalence of *Cryptosporidium* infection and identify associated risk factors among children under 5 years of age with diarrhea living in IDPs camps in the Daynile District of Mogadishu, Somalia. Establishing baseline data is essential to improve the understanding of the epidemiology of cryptosporidiosis in this high-risk population and inform future public health interventions and surveillance strategies.

## Methods

### Study area and setting

This study was conducted in IDPs camps located in the Daynile District of Mogadishu, Somalia. The camps are characterized by high population density, inadequate access to safe drinking water, limited sanitation facilities, and poor environmental hygiene. Healthcare services in the area are mainly provided through local health centers and non-governmental organizations that operate within or near the camps.

### Study design and period

A community-based cross-sectional study was conducted from December 2022 to August 2023 among children under 5 years of age residing in IDPs camps in the Daynile District of Mogadishu, Somalia.

### Study population and sampling

The study population consisted of children 5 years of age and below residing in IDPs camps in the Daynile District. Children presenting with diarrhea at the time of the survey or within the preceding 72 hours were eligible for stool sample collection. Diarrhea was defined as the passage of three or more loose or watery stools within a 24-hour period.

Participants were recruited using a convenience sampling approach from five IDPs camps in the Daynile District of Mogadishu, Somalia. Recruitment was conducted through community visits by the research team after obtaining permission from the camp leaders. Caregivers of eligible children were approached, informed about the objectives of the study, and invited to participate. Children who met the inclusion criteria and whose caregivers provided written informed consent were enrolled. To minimize clustering, only one eligible child was recruited from each household.

### Eligibility criteria

#### Inclusion criteria

Children 5 years of age or younger who resided in IDPs camps in the Daynile District of Mogadishu were included in the study. Eligible participants were children 5 years of age or younger residing in IDPs camps who were reported by their caregivers to have had diarrhea at the time of recruitment or within the preceding 72 hours. Stool consistency was assessed at the time of specimen collection according to the study definition of diarrhea.

#### Exclusion criteria

Children older than 5 years of age were excluded from the study. Children were excluded if their caregivers declined to provide written informed consent or if their age could not be reliably confirmed.

### Ethical considerations

Ethical approval was obtained from the Jamhuriya University Research Ethics Committee (JUREC), Faculty of Medicine and Health Science, Jamhuriya University of Science and Technology, Mogadishu, Somalia (approval number JUREC073/FMHS169/052022). Written informed consent was obtained from parents or legal guardians before enrollment in the study. The study objectives, procedures, potential risks, and benefits were explained in either Somali or English according to the caregiver’s preference. For caregivers who were unable to read or write, the consent information was explained verbally in Somali by a trained member of the research team and informed consent was obtained before participation. Participation was voluntary and the confidentiality of all participant information was strictly maintained.

### Data collection

A structured questionnaire developed in English and translated into Somali was used to collect sociodemographic, clinical, and hygiene-related information from parents or primary caregivers. The questionnaire was administered by trained members of the research team. For caregivers who were unable to read or write, the questions were explained in Somali and their responses were recorded by the interviewer.

### Stool sample collection

Fresh stool samples were collected from children with diarrhea using clean, leak-proof, and properly labeled plastic containers. Caregivers were instructed on the correct stool collection procedures. Samples were collected at the household level or health posts and transported to the parasitology laboratory of Jamhuriya University of Science and Technology within the same day of collection.

### Sample handling and preservation

Each stool specimen was examined macroscopically for consistency and adequacy. Samples were preserved in 10% formalin at room temperature to maintain oocyst morphology for microscopic examination. The use of formalin fixation limited downstream molecular analysis; therefore, no molecular assays were performed in this study and all diagnoses were based solely on microscopy.

### Laboratory diagnosis: modified Ziehl–Neelsen staining

Smears were prepared from preserved stool samples using wooden applicators on clean glass slides and allowed to air-dry. The smears were fixed in absolute methanol for 3 minutes, stained with strong carbol fuchsin for 15 minutes, and then rinsed with tap water. Decolorization was performed using 1% hydrochloric acid in ethanol for 15 seconds, followed by counterstaining with 1% methylene blue for 30 seconds. Slides were air-dried and examined under a light microscope.

### Microscopic examination and definition of positivity

Slides were examined using 40 × magnification for initial scanning and 100 × oil immersion for confirmation. A minimum of 50 microscopic fields per slide were examined before a slide was considered negative. A sample was defined as positive when one or more *Cryptosporidium* oocysts, appearing as round to ovoid, acid-fast, red-stained structures measuring approximately 4-6 μm, were observed. Unstained or poorly stained structures were not considered diagnostic.

### Quality control and observer agreement

All slides were examined independently by two trained laboratory technologists who were blinded to the clinical data. In cases of discordant results, the slides were jointly re-examined to reach a consensus. Positive and negative control slides were included periodically to ensure staining consistency and diagnostic accuracy.

### Oocyst load grading

For positive samples, oocyst burden was semi-quantitatively graded as follows:

1. 1+ (low): 1-10 oocysts per preparation,
2. 2+ (moderate): 11-50 oocysts per preparation,
3. 3+ (high): >50 oocysts per preparation.

### Statistical analysis

Data were entered and analyzed using SPSS version 21 (IBM Corp., Armonk, NY, USA). Descriptive statistics were used to summarize participant characteristics. Associations between *Cryptosporidium* infection and potential risk factors were assessed using chi-square tests and logistic regression. A *P* < 0.05 was considered statistically significant.

## Results

### Sociodemographic, hygiene, and clinical characteristics

A total of 384 children under 5 years of age were included in the study. Of these, 182 (47%) were male and 202 (53%) were female. The age distribution showed that 111 children (28.9%) were 2 years old, 150 (39.1%) were 3 years old, and 123 (32.0%) were 4 years old.

Most caregivers had no formal education (277 of 384, 72.1%), whereas 97 (25.3%) had completed primary education and 10 (2.6%) had secondary education. Regarding occupation, 231 (60.2%) caregivers were employed and 153 (39.8%) were unemployed.

With respect to sanitation, 350 (91.1%) households reported using pit latrines, whereas 34 (8.9%) households practiced open defecation. Children’s feces were mainly disposed of in toilets or latrines (347 of 384, 90.4%), whereas 37 (9.6%) households disposed of feces in the garbage. Only 16 (4.2%) households reported having access to soap for handwashing.

At the time of stool specimen collection, 372 (96.9%) children had diarrheal stools, whereas 12 (3.1%) children produced semi-formed or semi-solid stools that did not meet the study definition of diarrhea, although they had been enrolled based on caregiver-reported diarrhea at recruitment or within the preceding 72 hours.

Among these, 216 (56.3%) had one to three diarrheal episodes per day, 155 (40.4%) had four to six episodes, and 13 (3.4%) had more than six episodes per day. Abdominal pain was reported in 289 (75.3%) children, fever in 78 (20.3%), and vomiting in 17 (4.4%). Medical care for diarrheal illness was sought for 359 children (93.5%). Detailed characteristics are presented in Table 1.

**Table 1:** Sociodemographic, hygiene, and clinical characteristics of study participants (n = 384).

Characteristic	Category	Frequency	Percentage (%)
Age (years)	2	111	28.9
	3	150	39.1
	4	123	32.0
Sex	Male	182	47.4
	Female	202	52.6
Caregiver education	No formal education	277	72.1
	Primary	97	25.3
	Secondary	10	2.6
Caregiver occupation	Employed	231	60.2
	Unemployed	153	39.8
Toilet facility	Pit latrine	350	91.1
	Open defecation	34	8.9
Disposal of child’s feces	Toilet/latrine	347	90.4
	Garbage	37	9.6
Access to soap	Yes	16	4.2
	No	368	95.8
Diarrhea	Yes	372	96.9
	No	12	3.1
Diarrhea frequency/day	1-3 times	216	56.3
	4-6 times	155	40.4
	>6 times	13	3.4
Associated symptoms	Fever	78	20.3
	Abdominal pain	289	75.3
	Vomiting	17	4.4
Medical care sought	Yes	359	93.5
	No	25	6.5

### Prevalence of cryptosporidiosis and oocyst load

Of the 384 stool samples examined, 23 (6.0%) samples were positive for *Cryptosporidium* oocysts, whereas 361 (94.0%) samples were negative.

Among the 23 positive cases, 10 (2.6%) children had a low oocyst load (1+), eight children (2.1%) had a moderate oocyst load (2+), and five children (1.3%) had a high oocyst load (3+). The distribution of infection status and oocyst load is shown in Table 2.

**Table 2:** Prevalence of *Cryptosporidium* infection and oocyst load (n = 384).

Variable	Frequency	Percentage (%)
*Cryptosporidium* status		
Positive	23	6.0
Negative	361	94.0
Oocyst load among positives		
Low (1+: 1-10 oocysts)	10	2.6
Moderate (2+: 11-50 oocysts)	8	2.1
High (3+: >50 oocysts)	5	1.3

### Factors associated with Cryptosporidium infection

The multivariable logistic regression analysis showed that children whose caregivers had primary education had significantly lower odds of *Cryptosporidium* infection than those in the reference education category (adjusted odds ratio [AOR] = 0.08; 95% confidence interval [CI]: 0.01-0.75; *P* = 0.027). Children whose caregivers sought medical care for diarrheal illness also had significantly lower odds of infection (AOR = 0.28; 95% CI: 0.09-0.90; *P* = 0.032). No statistically significant associations were observed between *Cryptosporidium* infection and the child’s sex (*P* = 0.276) or caregiver occupation (*P* = 0.786). The full regression results are presented in Table 3.

**Table 3:** Factors associated with cryptosporidium infection among children under 5 years of age.

Variable	Category	AOR (95% CI)	*P*-value
**Sex**	Male	2.05 (0.56-7.49)	0.276
	Female (ref)	1.00	—
**Caregiver education**	No formal education	0.22 (0.04-1.28)	0.092
	Primary	0.08 (0.01-0.75)	0.027^a^
	Secondary (ref)	1.00	—
**Caregiver occupation**	Unemployed	1.20 (0.32-4.56)	0.786
	Employed (ref)	1.00	—
**Sought medical care**	Yes	0.28 (0.09-0.90)	0.032^a^
	No (ref)	1.00	—

Abbreviations: AOR, adjusted odds ratio; CI, confidence interval; ref, reference category.

a Statistically significant at *P* < 0.05.

## Discussion

This study investigated the prevalence of *Cryptosporidium* infection and associated risk factors among children under 5 years of age with diarrhea living in IDPs camps in the Daynile District of Mogadishu, Somalia. The overall prevalence of *Cryptosporidium* infection was 6.0%. This prevalence is lower than that reported in similar studies from Cameroon, Ethiopia, Sudan, Tanzania, Nigeria, and Malawi, where prevalence estimates among children with diarrhea ranged from approximately 8% to over 30% [11,12,14,[19], [20], [21]]. Differences in prevalence across studies may partly reflect variations in environmental conditions, sanitation practices, study populations, and diagnostic methods. Several of these studies used more sensitive diagnostic methods, including antigen detection assays or polymerase chain reaction, whereas the present study relied on modified Ziehl–Neelsen staining, which has lower sensitivity for detecting *Cryptosporidium* oocysts, particularly in cases with a low parasite burden [5,22]. Consequently, the true prevalence of *Cryptosporidium* infection in our study population may have been underestimated. This finding suggests that diarrheal illness in the study population is likely multifactorial [23,24]. Other enteric pathogens, such as bacteria, viruses, and non-*Cryptosporidium* parasites, were not investigated and may have contributed substantially to the burden of diarrhea. Therefore, the diarrheal symptoms observed in this study cannot be attributed solely to *Cryptosporidium* infection [1,8,12]. The reasons for this difference are not fully understood and may relate to behavioral, environmental, or cultural factors that influence exposure risk. However, given the observational nature of the study, these associations should be interpreted with caution.

This counterintuitive result may reflect reverse causality, whereby caregivers adopt improved hygiene practices in response to a child’s illness rather than as a preventive measure. Similar observations have been reported in other cross-sectional studies conducted in low-resource settings, highlighting the limitations of inferring temporal relationships from cross-sectional data.

Seeking medical care for diarrheal illness was associated with lower odds of *Cryptosporidium* infection. Although this finding does not imply a direct protective effect against infection, it may reflect earlier health care–seeking behavior, improved caregiver awareness, and timely management of diarrheal illness. The management of cryptosporidiosis is primarily supportive and includes oral rehydration, nutritional support, and correction of dehydration and electrolyte imbalance. Nitazoxanide may be effective in immunocompetent children; however, treatment can be more challenging in immunocompromised individuals, particularly those living with HIV [23,25,26]. Because HIV status was not assessed in the present study, we could not evaluate its influence on the occurrence or severity of cryptosporidiosis. These findings underscore the importance of accessible health care services for the early recognition and appropriate management of diarrheal diseases among children living in IDPs camps.

No significant associations were observed between *Cryptosporidium* infection and diarrhea frequency or other clinical symptoms, including fever, vomiting, and abdominal pain. These findings are consistent with previous studies indicating that clinical presentation alone is insufficient to distinguish cryptosporidiosis from other causes of childhood diarrhea. Therefore, laboratory confirmation remains essential for the accurate diagnosis of *Cryptosporidium* infection in children presenting with diarrhea to distinguish cryptosporidiosis from other causes of diarrhea, further emphasizing the importance of laboratory-based diagnosis [19,27,28].

This study has several limitations that should be acknowledged. First, the use of conventional microscopy may have resulted in underdiagnosis due to its limited sensitivity. Second, molecular characterization of *Cryptosporidium* species was not performed, preventing differentiation between zoonotic and anthroponotic transmission pathways. Third, the small number of positive cases in some exposure categories may have limited the precision of the estimated associations. In addition, although participants were enrolled based on caregiver-reported diarrhea or diarrhea within the preceding 72 hours, a small number of children produced semi-formed stools at the time of specimen collection and were classified as having non-diarrheal stools according to the study definition. This may have introduced a small degree of clinical misclassification. Finally, the cross-sectional design precludes causal inference.

Despite these limitations, the study provides important initial data on *Cryptosporidium* infection among children in Somali IDPs camps. The findings highlight the need for integrated public health interventions that strengthen WASH infrastructure and improve access to health care services in IDPs settings. This finding should be interpreted with caution because only a small number of caregivers had a secondary education [[29], [30], [31]]. Future studies should incorporate more sensitive diagnostic methods, investigate co-infections with other enteric pathogens, and explore species-level transmission dynamics to better inform targeted control strategies.

## Conclusion

To the best of our knowledge, this study provides the first documented evidence of *Cryptosporidium* infection among children under 5 years of age living in IDPs camps in Mogadishu, Somalia. The prevalence of cryptosporidiosis was 6.0% among children aged <5 years with diarrhea, indicating that *Cryptosporidium* is present in this high-risk population.

Several factors were statistically associated with infection, including male sex, caregiver unemployment, access to soap for handwashing, and health care–seeking behavior. These associations highlight the complex interplay between socioeconomic conditions, hygiene practices, and access to health care in displaced populations. However, the cross-sectional nature of the study does not allow causal inferences to be made.

Despite the relatively low prevalence of *Cryptosporidium*, the very high proportion of children experiencing diarrhea suggests that multiple enteric pathogens are likely contributing to the disease burden in IDPs camps. Because diagnosis relied on conventional microscopy, the true prevalence of infection may have been underestimated.

The findings underscore the need for integrated public health interventions that strengthen WASH infrastructure, improve access to health care services, and address underlying socioeconomic vulnerabilities in IDPs settings. Future research should incorporate more sensitive diagnostic methods, assess co-infections with other enteric pathogens, and explore species-level transmission dynamics to better inform targeted prevention and control strategies.

## Declaration of competing interest

The authors have no competing interests to declare.

## Funding

This research did not receive any specific grant from funding agencies in the public, commercial, or not-for-profit sectors. All study-related expenses were supported by the authors and conducted as part of institutional academic research at Jamhuriya University of Science and Technology, Mogadishu, Somalia.

## Ethical approval and consent to participate

Ethical approval was obtained from the JUREC, Faculty of Medicine and Health Sciences, Jamhuriya University of Science and Technology, Mogadishu, Somalia (approval number JUREC073/FMHS169/052022). Written informed consent was obtained from the parents or legal guardians of all participating children before their enrollment. For caregivers who were unable to read or write, the consent information was explained verbally in Somali by a trained member of the research team and informed consent was obtained before their participation. Participation was voluntary and the confidentiality of participants information was maintained.

## Consent for publication

Not applicable. No individually identifiable information or images of participants are presented in this manuscript.

## Availability of data and materials

The data sets generated and/or analyzed during the current study are available from the corresponding author on reasonable request, subject to applicable ethical and confidentiality requirements.

## CRediT authorship contribution statement

**Bashir Abdirahman Guled:** Conceptualization, Data curation, Formal analysis, Methodology, Writing – original draft. **Abdifetah Ibrahim Omar:** Supervision, Visualization. **Hussein Ibrahim Gabow:** Visualization, Writing – original draft. **Anwar Abdullahi Adam:** Data curation, Software. **Ifrah Ahmed Warsame:** Software, Validation, Visualization. **Hafza Jama Hayow:** Methodology, Project administration, Software, Validation. **Kauther Mohamed Omar:** Methodology, Writing – original draft, Writing – review & editing. **Omar Osman Mohamed:** Conceptualization, Data curation, Software. **Mohamed Khalif Abdulle:** Methodology, Software, Writing – original draft. **Ahmed Hassan Said:** Data curation, Formal analysis, Methodology. **Ahmed Saed Hussein:** Data curation, Formal analysis, Methodology.
